# Quantitative Measurement of Morphological Characteristics of PTFE Composite Transfer Films Based on Computer Graphics

**DOI:** 10.3390/ma16041688

**Published:** 2023-02-17

**Authors:** Yuan Qi, Bugong Sun, Honggang Wang, Yang Zhang, Gui Gao, Peng Zhang, Xiaobao Zheng

**Affiliations:** 1 Mechanical and Electronical Engineering College, Gansu Agricultural University, Lanzhou 730070, China; 2 State Key Laboratory of Solid Lubrication, Lanzhou Institute of Chemical Physics, Chinese Academy of Sciences, Lanzhou 730000, China

**Keywords:** PTFE, PEEK, Nano-ZrO_2_, tribological behavior, transfer film, morphological characteristics

## Abstract

In this paper, the tribological properties of polytetrafluoroethylene (PTFE) composites filled with Nano-ZrO_2_ and polyetheretherketone (PEEK) particles were tested for sliding friction against a counterpart metal (ASTM 1045 steel) using a linear reciprocating friction and wear experimental machine. Data on tribological performance and optical images of the transfer film were obtained at various friction stages for the material. MATLAB software was employed to develop quantitative analysis procedures for the morphological characteristics of the transfer film. The program enables image enhancement and morphological processing of transfer film images, then identifies, extracts, and quantifies the geometric and textural properties of the transfer film as a foundation for analyzing the variation of the characteristics and their relationship to the tribological properties of the material. The results demonstrated that the geometric, morphological, and textural characteristics of the transfer film were dynamically developing during the friction process, with noticeable differences between various friction stages and a significant impact on the tribological properties of the material. Quantitative analysis revealed a good correlation between the trends of some morphological and textural characteristics (the coverage, area, diameter, roundness, consistency, and texture entropy) of the transfer film and the wear resistance of the PTFE composite. Therefore, these morphological and textural characteristics can thus be used to quantify the transfer film quality and utilized as an indirect indicator of the tribological properties of the material.

## 1. Introduction

PTFE is a polymer material with a low coefficient of friction, corrosion resistance, and a wide range of operating temperatures, so it is often employed as a self-lubricating material and is commonly paired with metal materials to form sliding friction pairs, which are widely used in the field of industrial wear reduction and lubrication [1,2,3,4]. For the reason that the surface energy of the counterpart metal is higher than that of the polymer and the metal surface has microscopic concave and convex peaks, the relatively soft polymer friction surface is subject to abrasion by the hard metal surface during the friction process between the polymer and the counterpart metal, resulting in the detachment of some polymer material from the substrate and the formation of three-body frictional movements between the friction interfaces. Some of the material separated from the polymer matrix is thrown out of the friction system in the form of abrasive debris, while others are gradually deposited and adhere to the surface of the counterpart metal to form a thin film known as a “transfer film” [5,6,7,8,9,10]. This transfer film prevents direct contact and friction between the polymer and the counterpart metal surface, changing the microscopic environment between the surface interfaces of the sliding friction system and playing an important protective and lubricating role [11,12,13]. 

In recent years, the properties of transfer films have been extensively studied, and it was agreed that the firmness and homogeneity of the transfer film had a significant impact on the tribological properties of the polymeric material. The research found that the transfer film was in a dynamic state of change during the friction process and classified its evolution into three friction stages: the initiation stage, transition stage, and stabilization stage, according to the morphological characteristics of the transfer film. Physical and chemical testing methods were also used by researchers to quantify and analyze the thickness and morphological distribution patterns of transfer films. Urueña et al. [14] used an optical profilometer to measure the thickness of the PTFE composite transfer film and concluded that the volumetric wear rate during the stable wear phase is proportional to the cube of the transfer film thickness. Gosvami et al. [15] observed the in situ growth process of the transfer film using atomic force microscopy. Ye et al. [16,17] defined the free-space length (*L_f_*) to measure the gap between transfer films and established a mathematical model of the relationship between wear rate and free-space length. Christian et al. [18] used cyanoacrylate to bond the transfer film and copper sheet together and observed a correlation between bond strength and material wear rate, which decreased as the bond strength of the transfer film increased. Light and electron microscopy were frequently used by researchers studying transfer films to obtain microscopic images of their morphology, which were then characterized and analyzed [19,20,21,22]. Wahl et al. [23] observed and measured the thickness of the transfer film in situ using the Newtonian ring interferometric fringe method. Gu et al. [24] proposed a method for the quantitative testing of transfer films based on infrared transmission techniques and concluded that the thickness of the transfer film shows a linear relationship with the area of the infrared absorption peak of the transfer film at 1205 cm^−1^. Zhang et al. [25] used statistical methods to observe the characteristic values of different thicknesses of SiO_2_/PTFE composite transfer films and concluded that there is a strong correlation between the maximum thickness value of the transfer film and the wear rate of the material, with the wear rate decreasing as the maximum thickness of the transfer film increases.

Despite the extensive research conducted on the properties of transfer films, there currently needs to be an accurate and efficient quantitative system for evaluating the morphological characteristics of these films. This study aimed to address this gap by using an optical microscope to capture images of the transfer film of PTFE composites at various friction stages. Additionally, MATLAB software was utilized to develop a program that can automatically and accurately identify and extract morphological features of the transfer film based on computer graphics principles and quantitatively calculate the geometric characteristics (perimeter, area covered, coverage, circularity, rectangularity) and textural characteristics (mean, standard deviation, third order moments, consistency, texture entropy) of the transfer film. The research identified characteristic morphological parameters that have a good correlation with the tribological properties of PTFE composites and utilized them as evaluation indicators of the transfer film’s quality. This methodology provides a tool to investigate and evaluate the morphological characteristics of transfer films and aid in understanding their impact on tribological properties.

## 2. Materials and Methods

### 2.1. Preparation of PTFE Composites

The density of PTFE (POLYFLON M-18F, Daikin Corporation, Osaka, Japan) was 2.16 g/cm^3^, and the average particle size was 25 nm. Additionally, PEEK (Victrex PEEK 450 PF, Victrex Limited, Rotherham, UK) with a density of 1.3 g/cm^3^ and an average particle size of 50 nm was utilized. The ZrO_2_ nanoparticles (XFI01, Nanjing Xianfeng Nanomaterials Technology Co., Nanjing, China) used in the study had an average particle size of 20–40 nm, a density of 5.89 g/cm^3^, and a specific surface area of 130 m^2^/g.

The ZrO_2_ nanoparticles, PEEK particles, and PTFE powder were completely mixed mechanically using a high-speed airflow mixer according to the volume ratios (Nano-ZrO_2_ 5 vol.%, PEEK 10 vol.%, and PTFE 85 vol.%). The mixture was sieved through a 150-mesh sieve before being loaded into a specific mold. A hydraulic press was utilized to exert 45 MPa of pressure on the mold, after which the material was cold-pressed into the desired cylindrical shape. The pressed material was sintered into cylindrical specimens with a diameter of 6 mm within a furnace according to a pre-established temperature control program (sintering temperature set at 375 °C, heating rate of 2 °C/min, hold time of 120 min, and then cooled naturally to room temperature). The friction surface of the specimens was polished with 800-grit metallographic sandpaper for establishing adequate contact with the friction surface of the counterpart metal. The specimens were cleaned in an ultrasonic cleaner with acetone solvent, dried in a drying oven, weighed on an electronic balance (with an accuracy of 0.1 mg), and the data was recorded.

ASTM1045 was used as a counterpart metal substance to rub against PTFE composites, the surface of which was ground and polished to an average surface roughness of 0.005 μm to reduce the influence of roughness on the friction experiment results.

### 2.2. Friction and Wear Test

**Figure 1:**
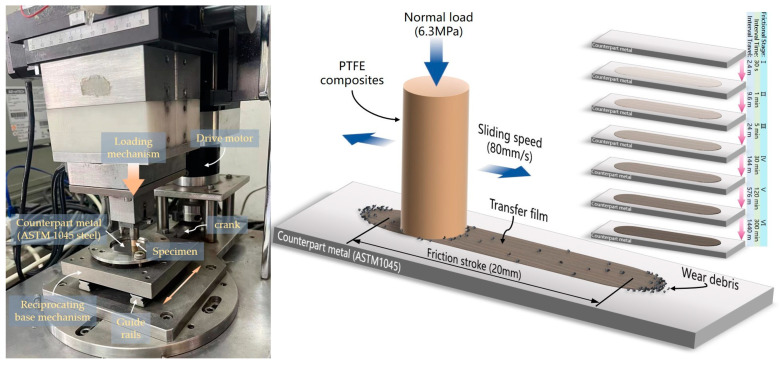
Schematic diagram of PTFE composite reciprocating friction test.

The tribological properties of the PTFE composite were tested using a linear reciprocating friction and wear tester (Model LSM-200, ZKKH Technology Development Co., Lanzhou, China), as illustrated in Figure 1. Before the friction test, the sample friction surface and the counterpart metal surface were cleaned with cotton balls bathed in acetone solvent. The specimen’s top was loaded with a normal load of 6.3 Mpa, the average sliding linear velocity was 80 mm/s, and the ambient temperature was 25 °C. The entire time for each specimen’s friction wear test was 456.5 min (a total stroke of 2191.2 m). The entire friction process was divided into six phases to evaluate the evolution of the tribological properties of the PTFE composite and the morphological characteristics of the transfer film at different friction stages (duration of each friction stage: 30 s, 1 min, 5 min, 30 min, 2 h, and 5 h). The sample and counterpart metal plate were carefully removed from the friction tester at the end of each stage of the friction test, and the following observations were made:

Weighing the specimen yielded mass loss, and the average volumetric wear rate *K* was computed at each stage of the friction test according to Equation (1).

(1) $$K=\frac{\Delta m}{\rho NL}$$

where ∆*m* is the wear weight, *ρ* is the specimen’s actual density, *N* is the normal load applied to the sample, and *L* is the total sliding distance.

**Figure 2:**
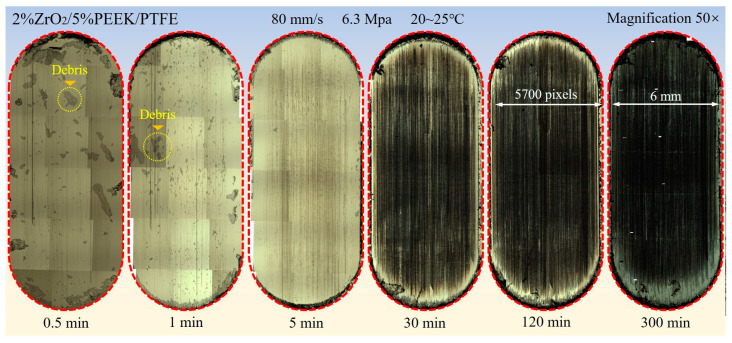
Synthetic image of the friction areas on the surface of the counterpart metal at different stages of friction.

An optical microscope (Axio Imager M2m, ZEISS, Jena, Germany) was used to capture high-resolution photographs of a partial area of the transfer film generated on the surface of the counterpart metal after each test stage (magnification of 50× to 1000×, image resolution of 2560 × 1920 pixels). The 50× magnified images of all partial areas of the transfer film were sequentially stitched together to form a single high-resolution image of the complete area of the transfer film, as shown in Figure 2.

Following completion of these operations, the sample and counterpart metal were replaced in situ on the friction tester for the next stage of the friction wear test. It was assured that the friction surface of the sample and the transfer film attached to the counterpart metal were not contaminated or damaged throughout disassembly, testing, and reassembly.

### 2.3. Pre-Processing of Transfer Film Images

In order to accurately segment the transfer film coverage area from the counterpart metal exposed area, it is necessary to preprocess the transfer film images to enhance the contrast between these two regions. The image preprocessing methods adopted in this study include image color space transformation, image edge smoothing, median filtering, and Laplacian sharpening.

#### 2.3.1. Grayscale Processing of Transfer Film Images

**Figure 3:**
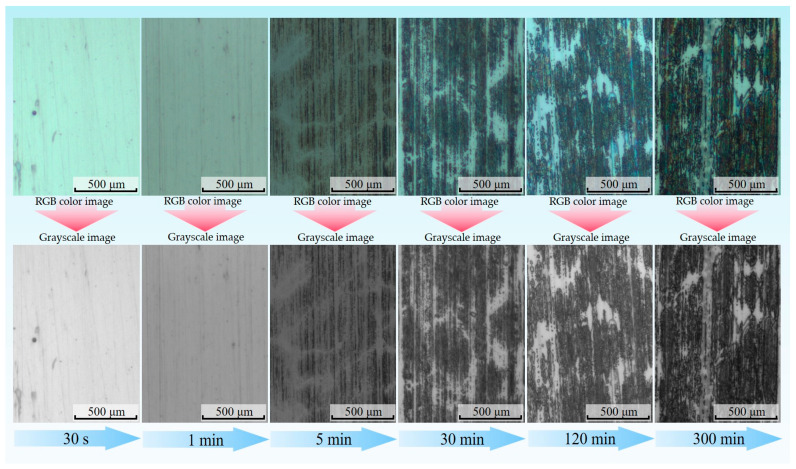
Greyscale image of the transfer film at different friction stages.

**Figure 4:**
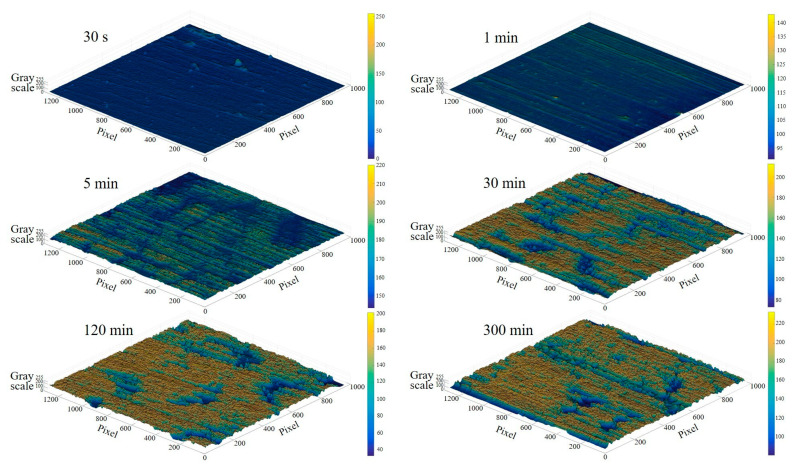
Grey scale diagram of the transfer film at different friction stages.

The photograph of the surface of a transfer film acquired by optical microscopy is a digital true color image in the RGB color mode. The RGB image is generated by superimposing scaled values (ranging from 0 to 255) on the three color channels R (Red), G (Green), and B (Blue), such that theoretically there are 256^3^ colors at each pixel point in the RGB image, thereby generating the three-dimensional color matrix of the image. RGB-format images of transfer film are computationally intensive to process and transmit, resulting in significant slowdowns in computing speed, and are susceptible to distortion and noise issues in localized image areas due to interfering factors. Using the rgb2gray function in MATLAB, the RGB image of the transfer film was converted to a greyscale image that contains only a single value of grey information (ranging from 0 to 255, with 0 representing black and 255 representing white). Figure 3 depicts the grayscale images of the transfer film at different friction stages. With the aid of the MATLAB visualization application, the 3D distribution of the different grey levels of the transfer film could be generated, providing a more comprehensible depiction of the distribution and evolution of the grey values of the transfer film at different friction stages, as shown in Figure 4. However, the relationship between the greyscale values and the true thickness of the transfer film needs to be further investigated in depth in later research work.

#### 2.3.2. Histogram Equalization of Transfer Film Grey-Scale Images

**Figure 5:**
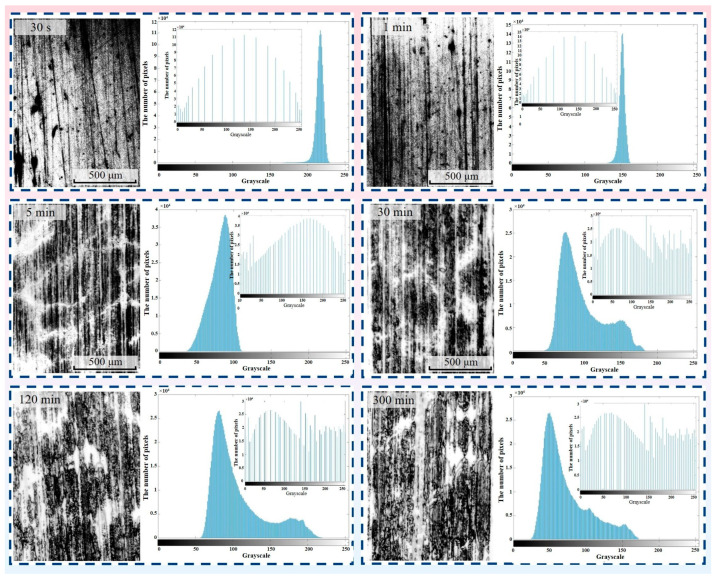
Histogram equalization of grey-scale images of transfer films.

As the contrast between the area covered by the transfer film and the bare metal in the greyscale image was relatively weak, the greyscale image required enhancement using a histogram equalization algorithm. The histogram equalization algorithm is based on the probability distribution of the grey levels in the image, utilizing the histogram equalization function in MATLAB, which is calculated to generate a new image with a balanced probability distribution of the grey levels, resulting in a maximization of the entropy value, thereby enhancing the global contrast of the transfer film image, as shown in Figure 5. The advantage of histogram equalization is that it is less computationally costly and can be inverted; nonetheless, it has the potential to increase the background noise of the metal substrate, requiring supplementary noise reduction measures to be applied to the image.

#### 2.3.3. Enhancement and Sharpening of Transfer Film Images

**Figure 6:**
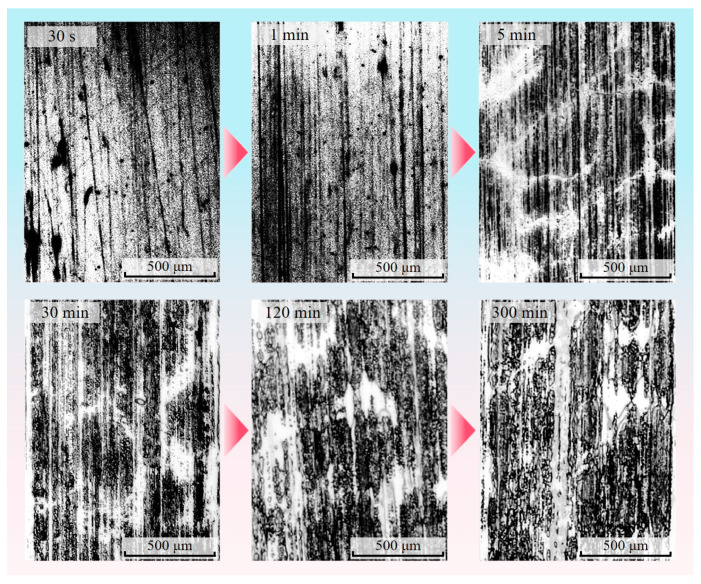
Transfer film image after median filtering and edge sharpening.

The medfilt2 function in MATLAB, based on the median filter principle, was used to reduce the noise of the histogram equalized transfer film greyscale image, preventing the blurring of image features caused by the linear filtering method. The median filtering method is a non-linear image enhancement technique based on the principle of arranging and counting the pixel points in the field according to their grey values, then calculating the median of these grey values and using this value as the new grey value of the central pixel point in the field. The Laplacian operator was then used to sharpen the edge contours of the transfer film, strengthening the edge information. The image of the transfer film after median filtering and edge sharpening is shown in Figure 6.

#### 2.3.4. Binarization of Transfer Film Grey-Scale Images

Based on the Otsu thresholding method, the image was dichotomized according to the grey-scale characteristics to distinguish between the area covered by the transfer film and the area of the counterpart metal backdrop. The Otsu method began by counting and normalizing the number of pixels in each grey level of the pre-processed transfer film greyscale image. A separation threshold T was pre-set in advance and used as a reference. The area in the grey-scale image within the range 0 ≤ grey-scale value ≤ T was then identified as the target area (the area covered by the transfer film), and the average grey-scale value *h*_0_ of the target area and the ratio *w*_0_ of the number of pixel points in the target area to the total number of pixels in the image were calculated. The area within the range T < grey-scale ≤ 255 was identified as the background area (the area where the pair of metals are exposed), and the average grey-scale value *h*_1_ of the background area and the number of pixels as a percentage of the total number of pixels in the image, *w*_1_, were calculated. According to Equation (2), the average greyscale value *h* for the entire domain of the transfer film greyscale image was calculated.

(2) $$h=w_{0}\times h_{0}+w_{1}\times h_{1}$$

The uniformity of the distribution of grey values in a transfer film image could be measured using the inter-class variance *c*, calculated as follows:

(3) $$c=2w_{0}\times \left(h_{0}-h\right)+2w_{1}\times \left(h_{1}-h\right)$$

**Figure 7:**
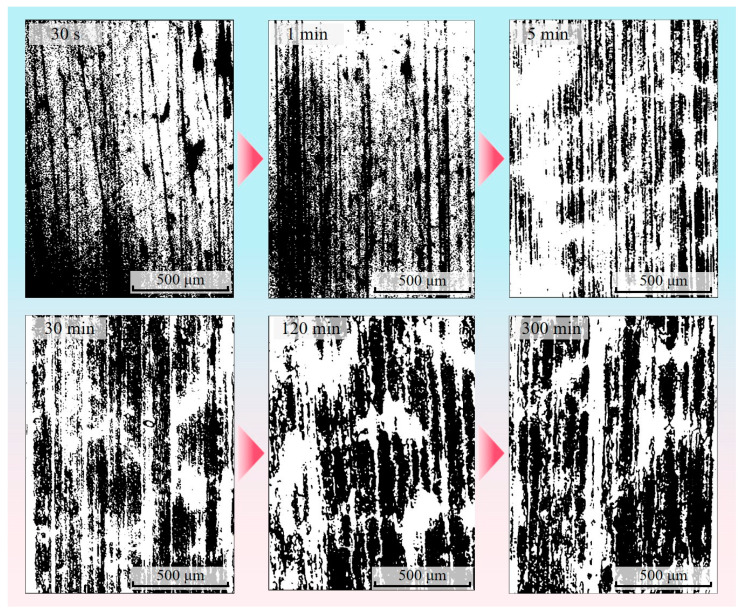
Binary images of the transfer film at different friction stages.

The image processing algorithm continuously adjusted the threshold value T and calculated the corresponding interclass variance value *c*. A larger *c* indicated a stronger contrast between the target area’s grey value and the background area’s grey value. By comparison, the largest inter-class variance value *c*_max_ was ultimately chosen, and the threshold T corresponding to it was deemed the optimal segmentation threshold T_opt_ for the transfer film image. The image processor assigned 0 to pixels in the range 0 ≤ grey-scale value ≤ T_opt_ (0 being black for areas covered by transfer film) and 255 to pixels in the range T_opt_ < grey-scale value ≤ 255. (255 being white for areas with bare metal). Figure 7 shows binary images of the transfer films obtained by the Otsu method at different friction stages, from which it can be inferred that there are virtually no transfer films on the counterpart metal surfaces obtained during the first and second stages of friction.

#### 2.3.5. Morphological Processing of Transfer Membrane Binary Images

**Figure 8:**
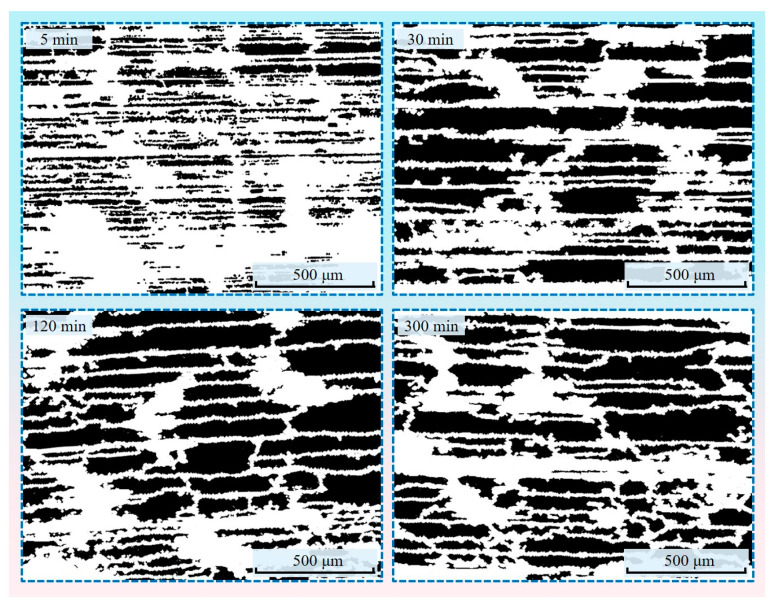
Binary images of the transfer film processed by morphology.

After converting the grayscale image of the transfer film to a binary image, there were holes and voids in some of the target areas, and some adjacent target areas were not entirely separated from each other. At the same time, there was also noise in the background areas. To increase the accuracy of the calculation of the transfer film characteristics, it was necessary to optimize further the binary image of the transfer film using morphological operations. The key to morphological processing was creating a structural element, also known as a probe, that was significantly smaller than the size of the target region. This probe traversed the entire binary image in an ordered manner, verifying through logical operations whether the target regions in the image could completely encompass the probe and determining the relationship between the various target regions and the background regions based on this. Four fundamental operations comprise morphological processing: expansion, erosion, open, and closed. The closed operation (expansion followed by erosion) smoothed the boundary by filling the target area’s holes and the boundary’s pits. The open operation (erosion followed by expansion) eliminated the target area’s protrusions to create a smooth boundary. For transfer film binary images with different morphological characteristics, these four operations were used reasonably to process the image, thus effectively improving the smoothness of the target area boundary and eliminating the noise in the background area. Since little transfer film was created on the surface of the counterpart metal during the first two friction stages (30 s and 1 min), only the binary images of the transfer film acquired during the last four friction stages (5 min, 30 min, 2 h, and 5 h) were morphologically processed, as shown in Figure 8.

### 2.4. Extraction and Quantification of Transfer Film Characteristics

After the morphological processing of the binary image of the transfer film, the features of the targeted area were extracted and quantified, including both the geometric shape feature parameters and the texture feature parameters.

#### 2.4.1. Quantification of Geometric Morphological Characteristics of Transfer Membranes

Geometric shape characteristics are global or local characteristics that describe the geometry of a transfer film. There are two categories of geometric shape characteristics: contour features, which describe the shape of an object’s boundary, and area features, which describe the shape of an object’s interior. The following five geometric shape feature parameters were extracted and quantified in this study:

(1) The perimeter *L* of the transfer film: the pixel points on the boundary of the transfer film were arranged in the counterclockwise direction and formed a closed boundary. The Euclidean distance d between two adjacent pixel points on the boundary was calculated using the 8-chain code method so that the perimeter *L* of the transfer film boundary was the sum of all *d*. The calculation formula is shown below:

(4) $$d=\sqrt{{\left(x_{1}-x_{2}\right)}^{2}+{\left(y_{1}-y_{2}\right)}^{2}}$$

(5) L=∑d

where *x* and *y* are the horizontal and vertical coordinate values, respectively, of the pixel point.

The 8-chain code method has several optimisation algorithms, and depending on the characteristics of the transfer film image, the gradient-based algorithm was chosen for this paper. This algorithm identifies the contours by analysing individual transfer film images’ gradient (derivative) values.

(2) The overall area *A*_1_ covered by the transfer film: each pixel in the target area was assigned a value of 1, whereas all pixels in the background area were assigned a value of 0. The sum of all the pixel points with a value of 1 was counted and used as the area covered by the transfer film. The calculation formula is shown below:

(6) $$A_{1}=\overset{m}{\underset{i=1}{\sum }}\overset{n}{\underset{j=1}{\sum }}I(x_{i},y_{i})$$

where *I* is the value of the pixel point.

(3) The coverage ratio of the transfer film *r*_cov_: the ratio of the transfer film’s overall coverage area *A*_1_ to the total number of image pixel points *A*. The calculation formula is shown below:

(7) $$r_{\mathrm{cov}}=\frac{A_{1}}{A}$$

(4) Transfer film roundness *C*: a measurement of how closely the transfer film’s boundary shape resembles a circle. A higher *C* value indicated that the shape of the transfer film was closer to a circle. The calculation formula is shown below:

(8) $$C=\frac{4\pi A_{\mathrm{ind}}}{L^{2}}$$

where *A*_ind_ is the area of a single individual transfer film.

(5) The squareness of the transfer film *R*: the ratio of an individual transfer film’s area to its smallest external rectangular area. *R* indicates the degree to which the shape of the transfer film resembles a rectangle. The calculation formula is shown below:

(9) $$R=\frac{A_{\mathrm{ind}}}{W\times H}$$

(10) $$W=\max\left(\sum_{j=1}^{n}I\left(x_{j},y_{j}\right)\right)$$

(11) $$H=\max\left(\sum_{i=1}^{m}I\left(x_{i},y_{i}\right)\right)$$

where *W* and *H* represent the width and height of individual transfer films, respectively.

#### 2.4.2. Quantification of Texture Characteristics of Transfer Membranes

Texture characteristics are a reflection of an image’s slowly changing or periodically varying structure. Texture characteristics demonstrate sequential repetition of local image structure, random arrangement of structural features, and roughly uniform consistency. On the basis of the statistical data provided by the grey scale histogram, it quantified and described the characteristic information of image textures in terms of interval distance, direction, magnitude of change, and speed of change by calculating the correlation of grey scale values between two points in an image at a particular distance and direction. The following five texture feature parameters were extracted and quantified in this study:

(1) The mean value of the transfer film *M*: the average luminance value of all pixel points in the target area, which is a measurement of the transfer film’s overall luminance. The calculation formula is shown below:

(12) $$M=\overset{G-1}{\underset{i=0}{\sum }}z_{i}p(z_{i})$$

where *z*_i_ is a random variable in the gray-scale value of the transferred film image; *G* is the gray-scale level in the image, and *p*(*z*_i_) is the gray-scale histogram corresponding to *z*_i_ in the image (*i* = 0, 1,…, *G* − 1).

(2) The standard deviation of the transfer film *SV*: the deviation of the pixel values in the area covered by the transfer film relative to the mean value. The larger the standard deviation value, the greater the range of variation of the grey values in the image. The calculation formula is shown below:

(13) $$SV=\sqrt{\mu_{2}(z)}$$

(14) $$\mu_{2}=\sum_{i=0}^{G-1}{\left(z_{i}-M\right)}^{2}p\left(z_{i}\right)$$

where *μ*_2_ is the second order moment of the mean value *M* of the transfer film.

(3) The third-order moment of the transfer film *μ*_3_: it quantifies the degree of deflection of the greyscale histogram and thus determines the symmetry of the histogram. The calculation formula is shown below:

(15) $$\mu_{3}=\sum_{i=0}^{G-1}{\left(z_{i}-M\right)}^{3}p\left(z_{i}\right)$$

(4) The consistency of the transfer film *U*: it reflects the texture’s smoothness in the transfer film image. The greater the consistency value, the smoother the image’s texture; conversely, the lower the consistency value, the rougher the texture. The calculation formula is shown below:

(16) $$U=\overset{G-1}{\underset{i=0}{\sum }}p^{2}(z_{i})$$

(5) The textural entropy of the transfer film *E*: it reflects the variability and complexity of the texture in the transfer film image. A higher entropy value *E* indicates a more complex transfer film shape; conversely, a lower entropy value *E* indicates a less chaotic transfer film shape. The calculation formula is shown below:

(17) $$E=-\overset{G-1}{\underset{i=0}{\sum }}p(z_{i})\log_{2}p(z_{i})$$

## 3. Results and Discussion

### 3.1. Evolution of Tribological Properties

**Figure 9:**
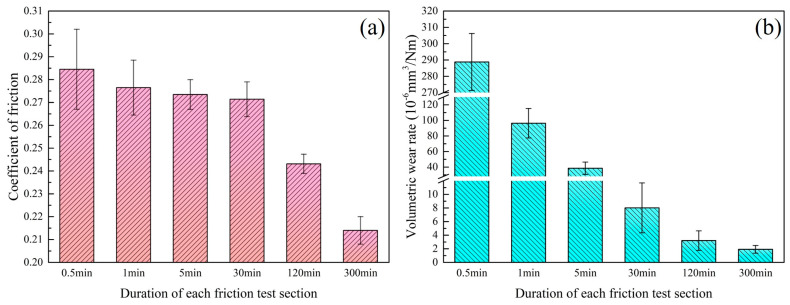
Tribological properties of Nano-ZrO_2_/PEEK/PTFE at different friction stages. (**a**) coefficient of friction; (**b**) volume wear rate.

The tribological properties of Nano-ZrO_2_/PEEK/PTFE were tested on a reciprocating friction and wear tester. Figure 9 depicts the tribological properties of the specimens at various stages of testing, demonstrating that the coefficient of friction and volumetric wear rate gradually decreased throughout the friction process and that this trend was closely related to the formation and evolution of the transfer film. Within the first three friction phases, there was a trend in the volumetric wear rate toward a significant decrease. Figure 3 and Figure 6 show that during the first friction phase (0.5 min), there was almost no transfer film on the counterpart metal surface, and only a small amount of the larger-sized abrasive debris was scattered and adhered to the friction surface. The lack of lubrication from the transfer film resulted in a volumetric wear rate of up to 288 × 10^−6^ mm^3^/Nm in this friction phase. The volumetric wear rate in the third friction stage (5 min) was significantly reduced to 38 × 10^−6^ mm^3^/Nm. At this stage, no abrasive debris was adhering to the counterpart metal’s surface, and a finer band of transfer film had formed, but the overall coverage area was insufficient. This phenomenon was caused by the rolling motion of the hard abrasive chips at the friction interface, which scraped the thinner transfer film, destroying the transfer film’s integrity. The thickness of the transfer film gradually increased in the stable friction stage, improving the solidity of the film and its ability to resist scraping by hard abrasive debris, promoting fusion between the transfer films, and increasing the coverage area so that the volumetric wear rate also decreased from 8 × 10^−6^ mm^3^/Nm in stage 4 (30 min) to 1.9 × 10^−6^ mm^3^/Nm in stage 6 (300 min), which was a 151-fold increase in wear resistance compared with stage 1. 

The coefficient of friction of the material fluctuated dramatically during the first four friction stages, with the average coefficient of friction remaining above 0.27 in each stage and exhibiting a more gradual overall reduction trend. The higher coefficient of friction was attributed to the incomplete coverage and protection of the surface of the counterpart metal by the transfer film, the exposed microscopic metal bumpy peaks, and the hard abrasive debris at the friction interface that would scratch the surface layer of the specimen. The complete and homogeneous transfer film provided lubrication and protection in the fifth and sixth friction stages, resulting in a significant reduction in the average coefficient of friction, which was 0.21 in stage 6.

### 3.2. Evolution of Morphological and Textural Features of Transfer Film

**Figure 10:**
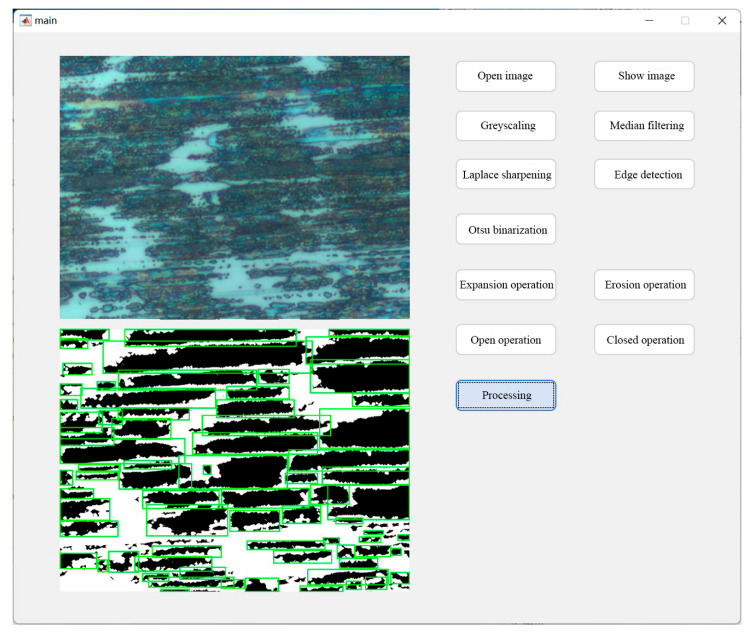
MATLAB program interface for transfer film feature identification and parameter extraction.

The optical images of the transfer film at various friction stages were imported into the MATLAB program, as shown in Figure 10. By calculating the edge length of a single pixel in the original 1000× magnified transfer film image (resolution 1280 × 960) as 0.05263 μm, the length and width of this image are therefore 67.37 μm and 50.5 μm respectively. For calculation purposes, the resolution of the image is adjusted to 2700 × 2025, giving an edge length of 25 nm for a single pixel and an area of 625 nm^2^.The program recognized and extracted the transfer film, as well as quantified its geometrical and textural features. The final output could be used as a database to study the evolution of transfer film features.

**Figure 11:**
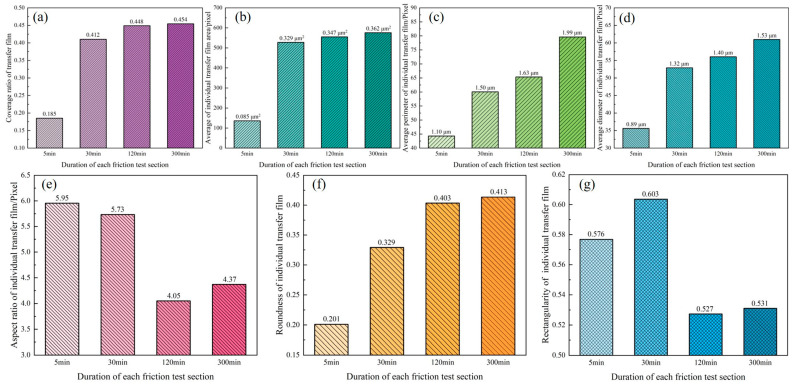
Geometry characteristics of transfer film. (**a**) coverage ratio; (**b**) area; (**c**) perimeter; (**d**) diameter; (**e**) aspect ratio; (**f**) roundness; (**g**) rectangularity.

Figure 11 depicts the evolution of the geometrical characteristics of the transfer film during the four stages (5 min, 30 min, 2 h, and 5 h) of the late friction test. When compared with the previous friction stage, the overall coverage of the transfer film (Figure 11a) and the area of the individual transfer film (Figure 11b) increased significantly within the fourth friction stage, after which the growth trend tapered off, consistent with the transfer film shown in the optical microscopy images. The gradual increase in the boundary perimeters (Figure 11c) and diameters (Figure 11d) of the transfer films indicated that the individual transfer films were also growing in size. The aspect ratio of the transfer film (Figure 11e) decreased significantly in friction stages 5 and 6 compared with previous friction stages, indicating that the transfer film gradually fused and developed from its initial banded morphology to a blocky morphology. During the friction process, the value of circularity of the transfer film (Figure 11f) gradually increased, indicating that the boundary profile of the transfer film approaches a circle more closely; however, the rectangularity of the transfer film (Figure 11g) did not exhibit a significant trend. 

**Figure 12:**
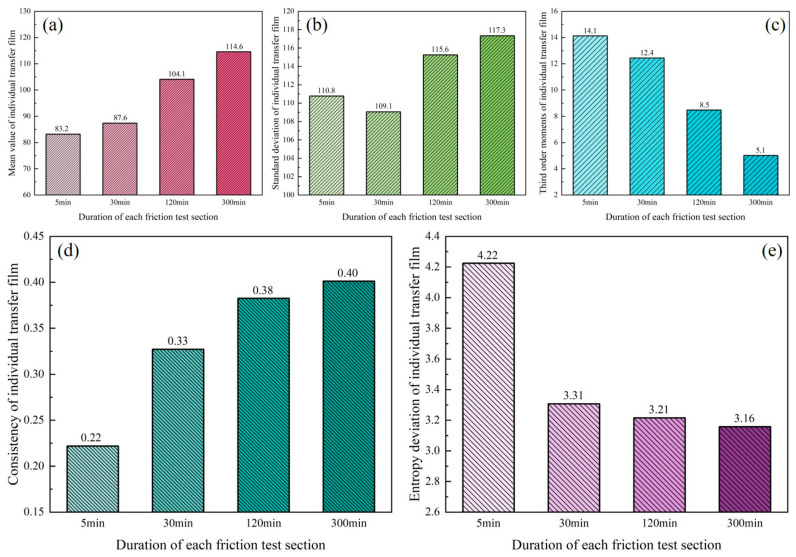
Textural characteristics of the transfer film parameters. (**a**) mean value; (**b**) standard deviation; (**c**) third-order moments; (**d**) consistency; (**e**) texture entropy.

The evolution of the transfer film’s textural characteristics is depicted in Figure 12, which reveals that the mean (Figure 12a) and consistency (Figure 12d) of the transfer film exhibited a trend of gradual increase, in contrast to the third-order moment (Figure 12c) and texture entropy (Figure 12e) of the transfer film, which exhibited a trend of gradual decrease, while the standard deviation (Figure 12b) of the transfer film did not exhibit a clear pattern of change. As the friction test progressed, the increased consistency of the transfer film indicated that the texture of the transfer film gradually transitioned from rough to smooth. In contrast, the decrease in texture entropy indicated a gradual reduction in the image texture complexity of the transfer film.

### 3.3. Correlation between Tribological Properties and Transfer Film Characteristic Parameters

Using the Pearson correlation principle, the correlation between the morphological characteristics of the transfer film obtained at different friction stages and the tribological performance (volumetric wear rate and coefficient of friction) was analyzed. The formula for Pearson correlation is as follows:

(18) $$r=\frac{\sum_{i-1}^{n}\left(x_{i}-\bar{x}\right)\left(y_{i}-\bar{y}\right)}{\sqrt{\sum_{i-1}^{n}{\left(x_{i}-\bar{x}\right)}^{2}\sum_{i-1}^{n}{\left(y_{i}-\bar{y}\right)}^{2}}}$$

where *x* is the morphological characteristics of the transfer film; *y* is the tribological properties of the specimens at various friction stages, and *r* is the correlation coefficient between *x* and *y*. |*r*| > 0.95 is a significant correlation, |r| ≥ 0.8 is a high correlation, 0.5 ≤ |r| < 0.8 is a moderate correlation, 0.3 ≤ |r| < 0.5 is a low correlation, and |r| < 0.3 is no correlation. 

The results of the calculations are shown in Table 1, where it can be seen that the volume wear rate at different friction stages exhibited a significant correlation with the overall coverage of the transfer film, the size of the area of the individual transfer film, its diameter, roundness, consistency, and texture entropy, indicating that the greater the coverage of the transfer film on the surface of the counterpart metal, the smoother the borders, and the less chaotic the texture, the more it contributes to the wear resistance of the PTFE composite. In contrast, the coefficient of friction demonstrated significant correlations with only three textural features of the transfer film, namely the mean, standard deviation, and third-order moment, while the correlation with the geometrical features of the transfer film was relatively weak.

## 4. Conclusions

In this study, numerous characteristics of the transfer film were extracted and analyzed using a MATLAB-developed image feature quantification algorithm. The correlation mechanism between the tribological properties of Nano-ZrO_2_/PEEK/PTFE at different friction stages and the characteristics of the transfer film were investigated. Several of the drawn conclusions were as follows:

1. Throughout the friction process of Nano-ZrO_2_/PEEK/PTFE, abrasive debris attached to the counter metal’s surface and progressively accumulated to generate a transfer film. As the area covered by the transfer film and its thickness steadily increased, the volume wear rate of Nano-ZrO_2_/PEEK/PTFE decreased significantly, from 288 × 10^−6^ mm^3^/Nm in the first stage to 1.9 × 10^−6^ mm^3^/Nm in the sixth stage, and the average friction coefficient in each phase also showed a moderate but steady decrease. This suggests that the tribological properties of the PTFE composites are closely related to the quality and evolution of the transfer film.
2. A MATLAB-built program for transfer film feature quantization enabled the enhancement of the transfer film image and the reduction in noise in the image. Based on the Otsu algorithm and morphological principles, the transfer film area might be separated from the bare metal surface region with greater precision, and the transfer film’s edges could be recognized and extracted.
3. The results of the extraction and quantification of the geometric and textural features of the transfer film showed that the overall coverage ratio, area, perimeter, diameter, circularity, mean, and consistency characteristics of the transfer film demonstrated a gradual increase in dynamic evolution during the friction process, whereas the third-order moments and texture entropy demonstrated a trend of gradual decrease.
4. An analysis of the correlation between tribological properties and transfer film characteristics revealed that the volumetric wear rate exhibited significant correlations with six characteristic parameters, namely the coverage, area, diameter, roundness, consistency, and texture entropy of the transfer film, and therefore these six characteristic parameters can be used as a quantitative basis for evaluating the quality of the transfer film and the wear resistance of Nano-ZrO_2_/PEEK/PTFE. The algorithm will be further improved to increase the accuracy and efficiency of the identification of morphological features of the transfer film, and an attempt will be made to use quantitative analysis of the transfer film quality to determine the tribological properties of the material.

## Figures and Tables

**Table 1:** Correlation of transfer film morphological characteristics parameters with tribological properties (coefficient of friction and volumetric wear rate).

Morphological Characteristics of the Transfer Film		Correlation Coefficient with Volumetric Wear Rate (*r*)	Correlation Coefficient with Friction Coefficient (*r*)
Geometrical characteristics	Overall coverage ratio	−0.999910786	−0.658187884
	The area of the individual transfer film	−0.997891648	−0.623471999
	Perimeter	−0.886632773	−0.910310874
	Diameter	−0.983666792	−0.772315698
	Aspect ratio	0.746834732	0.837089055
	Roundness	−0.970752224	−0.798297282
	Rectangularity	0.446194053	0.837144576
Textural features	Mean value	−0.750643713	−0.986448891
	Standard deviation	−0.538869718	−0.955441271
	Third−order moments	0.771298518	0.987053339
	Consistency	−0.969531143	−0.816409685
	Texture entropy	0.999192918	0.644833901

## Data Availability

Not applicable.
